# Outcomes of patients with altered level of consciousness and abnormal electroencephalogram: A retrospective cohort study

**DOI:** 10.1371/journal.pone.0184050

**Published:** 2017-09-08

**Authors:** Paula Rodrigues Sanches, Thiago Domingos Corrêa, Taissa Ferrari-Marinho, Pedro Vicente Ferreira Naves, Carol Ladeia-Frota, Luís Otávio Caboclo

**Affiliations:** 1 Intensive Care Unit, Hospital Israelita Albert Einstein, São Paulo, Brazil; 2 Department of Clinical Neurophysiology, Hospital Israelita Albert Einstein, São Paulo, Brazil; University of Modena and Reggio Emilia, ITALY

## Abstract

**Introduction:**

Nonconvulsive seizures (NCS) are frequent in hospitalized patients and may further aggravate injury in the already damaged brain, potentially worsening outcomes in encephalopathic patients. Therefore, both early seizure recognition and treatment have been advocated to prevent further neurological damage.

**Objective:**

Evaluate the main EEG patterns seen in patients with impaired consciousness and address the effect of treatment with antiepileptic drugs (AEDs), continuous intravenous anesthetic drugs (IVADs), or the combination of both, on outcomes.

**Methods:**

This was a single center retrospective cohort study conducted in a private, tertiary care hospital. Consecutive adult patients with altered consciousness submitted to a routine EEG between January 2008 and February 2011 were included in this study. Based on EEG pattern, patients were assigned to one of three groups: Group Interictal Patterns (IP; EEG showing only interictal epileptiform discharges or triphasic waves), Group Rhythmic and Periodic Patterns (RPP; at least one EEG with rhythmic or periodic patterns), and Group Ictal (Ictal; at least one EEG showing ictal pattern). Groups were compared in terms of administered antiepileptic treatment and frequency of unfavorable outcomes (modified Rankin scale ≥3 and in-hospital mortality).

**Results:**

Two hundred and six patients (475 EEGs) were included in this analysis. Interictal pattern was observed in 35.4% (73/206) of patients, RPP in 53.4% (110/206) and ictal in 11.2% (23/206) of patients. Treatment with AEDs, IVADs or a combination of both was administered in half of the patients. While all Ictal group patients received treatment (AEDs or IVADs), only 24/73 (32.9%) IP group patients and 55/108 (50.9%) RPP group patients were treated (p<0.001). Hospital length of stay (LOS) and frequency of unfavorable outcomes did not differ among the groups. In-hospital mortality was higher in IVADs treated RPP patients compared to AEDs treated RPP patients [11/19 (57.9%) vs. 11/36 (30.6%) patients, respectively, p = 0.049]. Hospital LOS, in-hospital mortality and frequency of unfavorable outcomes did not differ between Ictal patients treated exclusively with AEDs or IVADs.

**Conclusion:**

In patients with acute altered consciousness and abnormal routine EEG, antiepileptic treatment did not improve outcomes regardless of the presence of periodic, rhythmic or ictal EEG patterns.

## Introduction

Approximately 5% of patients admitted to emergency department (ED) have altered states of consciousness and almost 1% are in coma [1]. Moreover, nearly 7% of adult patients admitted to the intensive care unit (ICU) have altered level of consciousness as a primary reason for ICU admission [2] while one in eight patients develop altered state of consciousness during ICU stay [3].

Very often, mental confusion, depressed level of consciousness and coma are caused by nonconvulsive seizures (NCS) and nonconvulsive status epilepticus (NCSE) [4]. Nonconvulsive seizures can be as frequent as 8% in comatose patients without signs of seizures activity [5]. Moreover, seizures further aggravate injury in the already damaged brain [6,7], potentially worsening outcomes in critically ill patients [8–10]. Thus, early seizures recognition through electroencephalography (EEG) monitoring and treatment have been advocated aiming at preventing further neurological deterioration [11].

Rhythmic and periodic patterns (RPP) are recognized as ictal-interictal uncertain EEG patterns [12]. They can represent an epiphenomenon of an injured brain, an interictal state or rather an ictal event [13]. However, patients with RPP on EEG can be diagnosed as NCSE if they have one of the following criteria: subtle clinical ictal phenomena, typical spatiotemporal evolution or response to antiepileptic treatment [14]. Nevertheless, so far it has been unclear if patients presenting with impaired consciousness and RPP patterns should be treated [12] as NCSE. Furthermore, the drugs commonly used to treat NCSE patients may have deleterious effects, such as increased risk of infection and death [15–17].

We hypothesized that rhythmic and periodic patterns are frequent in EEGs of patients presenting with acute consciousness impairment, and that antiepileptic treatment can affect their outcomes.

## Objective

Our objective was to evaluate the main patterns of routine-EEGs performed in patients with altered consciousness and to address the effect of antiepileptic treatment on modified Rankin Scale at hospital discharge [18] and on in-hospital mortality in adult patients with impaired level of consciousness and abnormal EEG.

## Materials and methods

### Design and setting

The study was approved by the ethics committee of Hospital Israelita Albert Einstein (Approval number 43474215.1.0000.0071) and written informed consent was waived. This was a single center retrospective cohort study conducted in a private, tertiary care hospital in São Paulo, Brazil.

### Patients

Consecutive adult (≥18 years) patients with altered consciousness submitted to a routine EEG between January 2008 and February 2011 were included in this study. These patients were in the ICU, step down unit or floor when their EEG was recorded. Indications for EEG performing included ‘alteration of consciousness’, ‘coma’, ‘acute mental confusion’, ‘encephalopathy’, ‘encephalitis’, and ‘status epilepticus’.

### Data collection and study variables

Study data were retrieved from patients’ digital medical records. Collected variables included demographics, comorbidities, level of consciousness when EEG was performed, clinical diagnosis of altered state of consciousness, EEG pattern, radiologic diagnosis by computer tomography (CT) or nuclear magnetic resonance image (RMI), administered treatments, modified Rankin Scale [18] at hospital discharge, hospital length stay (LOS) and in-hospital mortality. In patients admitted to the ICU, the need and duration of mechanical ventilation and ICU LOS were additionally collected.

### Antiepileptic treatment

Treatment decisions were made by consensus between on-duty intensivist and attending neurologists in the ICU, while in step down units and wards they were made by attending physicians.

Antiepileptic therapy was administered following international guidelines [19,20]. Briefly, first-line AEDs (intravenous bolus of diazepam or midazolam), with the objective of immediate seizure interruption when necessary, followed by second-line AEDs (one of the following: phenytoin, phenobarbital or valproic acid) when seizures persisted. Second-line AEDs could be administered intravenously or enterally. Third-line antiepileptic treatment was administered if no clinical or EEG improvement was observed with second-line AEDs treatment. Continuous intravenous anesthetic drugs (IVADs) as midazolam, propofol or barbiturates were initiated as third-line treatment. Barbiturates were used only if seizures persisted after midazolam or propofol use. We accessed administered AEDs and IVADs.

### EEG acquisition and analysis

Routine-EEG recording followed the requirements for performing clinical electroencephalography from the American Clinical Neurophysiology Society (ACNS) guideline [21]. Accordingly, all 21 electrodes were placed in the 10–20 System standard, and the instrument settings (sensitivities, filters, paper speed, and montage) agree with the guideline recommendations [21]. Additionally, simultaneous video recordings were performed to allow interpretation of clinical events and artifacts. Each EEG recorded contained 20 to 30 minutes of a technically satisfactory recording. All EEGs were performed at the bedside with the same technical standard, whether the patient was in the ICU, step-down unit or the floor.

All EEGs were analyzed independently by two certified clinical neurophysiologists with expertise in ICU EEG. Electroencephalogram patterns were described based on the glossary of the International Federation of Clinical Neurophysiology [22] and the American Clinical Neurophysiology Society (ACNS) Standardized Critical Care EEG Terminology [23]. Nonconvulsive seizures were defined according to criteria established by Young and cols. [24] and NCSE following Beniczky and cols. [25]. Whenever disagreement on EEG pattern occurred, consensus was obtained in a meeting with a third neurophysiologist. Normal EEGs were excluded.

Based on their EEG patterns, all patients were assigned to one of the following three groups: Interictal Patterns (IP; EEG showing rhythmic interictal epileptiform discharges or triphasic waves), Rhythmic and Periodic Patterns [RPP; at least one EEG with rhythmic or periodic patterns, including rhythmic delta activity (RDA), lateralized periodic discharges (LPD), bilateral independent periodic discharges (BIPD) and generalized periodic discharges (GPD)] and Ictal (at least one EEG with ictal patterns, such as electrographic seizures or status epilepticus).

### Statistical analysis

Categorical variables were displayed as absolute and relative frequencies. Numerical variables were presented as mean and standard deviation (SD) or median with interquartile range (IQR) in case of non-normal distribution, tested with Kolmogorov-Smirnov test.

Agreement between two raters (Interrater reability) on EEG analysis was accessed using kappa statistics. Comparisons between groups using chi-square test or Fisher exact test were made when appropriate. Continuous variables were compared using independent samples t-test or one-way analysis of variance (ANOVA) for normally distributed variables and Mann-Whitney U-test or Kruskal Wallis test in case of non-normal distribution. Post hoc comparisons after ANOVA or Kruskal Wallis test were performed using Tukey post-hoc test or Mann-Whitney U-test, respectively. To account for multiple comparisons, a Bonferroni correction was applied.

Unadjusted and adjusted logistic regression analyses were performed to address the impact of treatment on unfavorable outcomes (modified Rankin scale ≥3 and in-hospital mortality). Results were presented as odds ratio (OR) with 95% confidence interval (95%CI).

Two-tailed tests were used and when p<0.05, the test was considered statistically significant. The SPSS^™^ (IBM^™^ Statistical Package for the Social Science version 22.0 for Windows) was used for statistical analyses.

## Results

### Cohort included

**Fig 1:**
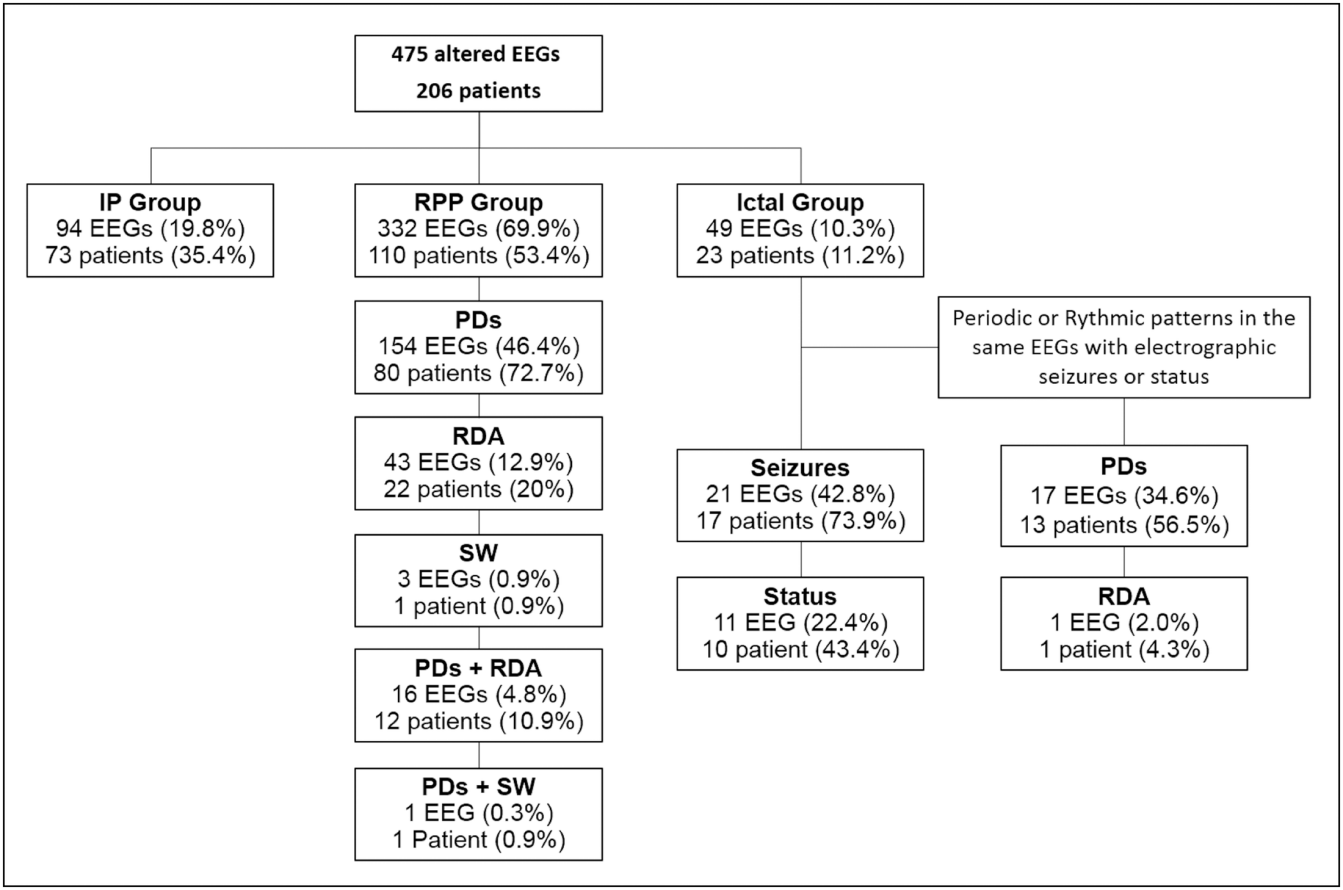
Flowchart of EEG analysis. IP group: patients with triphasic waves or interictal discharges. RPP group: patients with periodic or rhythmic patterns on EEG, according to American Clinical Neurophysiology Society Standardized Critical Care EEG Terminology [23]. Ictal group: patients with electrographic crisis or status on EEG. PD: periodic discharge. RDA: rhythmic delta activity. SW: spike-and-wave pattern.

**Fig 2:**
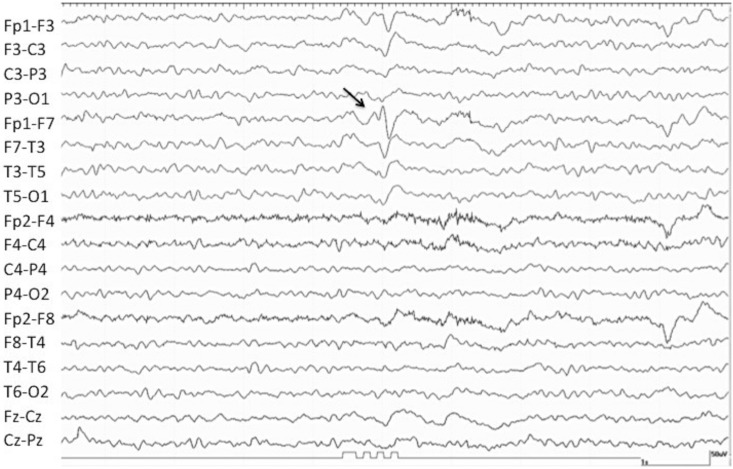
EEG recording of a 78-year-old woman with altered consciousness after a traumatic brain injury. The EEG shows a mild diffuse slowing of background rhythms, more pronounced over the left hemisphere, and a sharp wave over the anterior regions of this hemisphere (arrow). This patient was classified into Interictal Patterns group (IP group) due to the interictal pattern shown on her EEG.

**Fig 3:**
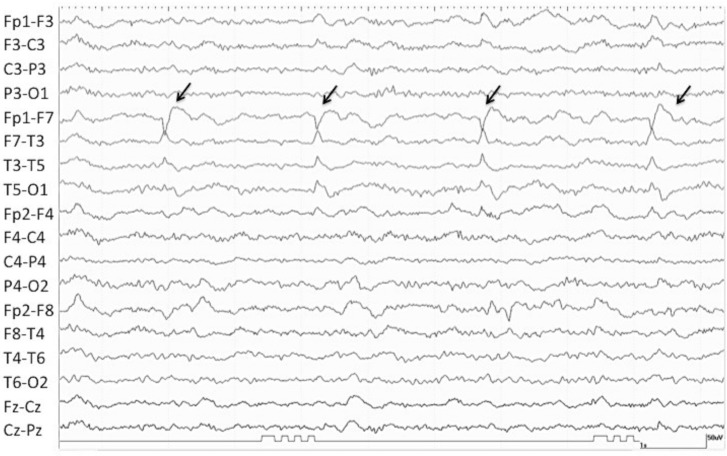
EEG recording of a 58-year-old man with herpes encephalitis presenting obtundation. The arrows point to periodic sharp waves over the anterior left temporal region (LPD—lateralized periodic discharges). The patient was enrolled to the Rhythmic and Periodic Patterns group (RPP group) due to the periodic pattern seen on his EEG.

**Fig 4:**
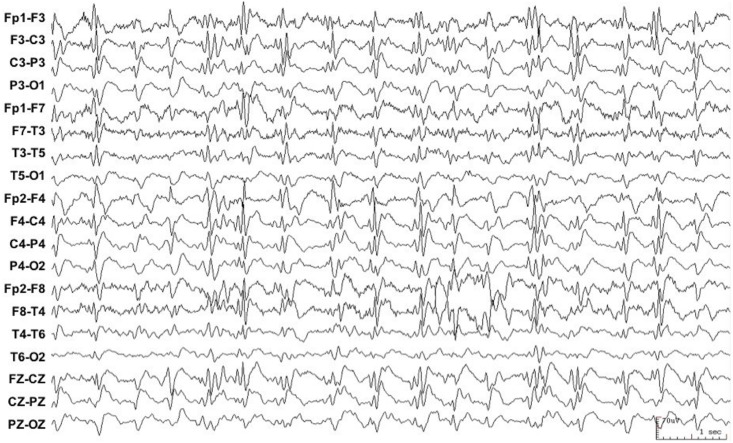
EEG recording of a 61-year-old woman in a coma state, after termination of a generalized tonic-clonic seizure. The EEG reveals continuous epileptiform discharges—sharp waves—over both right and left hemispheres, which in association with the clinical picture constitute a nonconvulsive status epilepticus. The patient was classified into Ictal group.

Between January 7, 2008 and February 5, 2011, 3,596 EEGs were performed, of which 1,364 were for consciousness impairment investigation. After exclusion of normal EEGs, and of patients with incomplete data or under eighteen, 475 EEGs from 206 patients were included in the final analysis (Fig 1). Interictal pattern was observed in 35.4% (73/206) of patients, RPP in 53.4% (110/206) and ictal pattern in 11.2% (110/220) of patients (Fig 1). Baseline characteristics of study patients are shown in Table 1. A representative EEG of IP, RPP and ictal groups are presented, respectively, in Figs 2, 3 and 4.

**Table 1:** Baseline characteristics of 206 patients with altered consciousness. Values represent median (IQR) or No. /Total No. (%).

Characteristics	All N = 206 (100.0)	IP N = 73 (35.4)	RPP N = 110 (53.4)	Ictal N = 23 (11.2)	P value*
Age, years	78 (65–86)	88 (70–88)	78 (65–84)	66 (55–75)^#^^&^	0.003^a^
Female	108 (52.4)	40 (54.8)	56 (50.9)	12 (52.2)	0.875^b^
Comorbidities					
Systemic hypertension	62 (30.1)	21 (28.8)	23 (30.9)	7 (30.4)	0.975^b^
Dementia	60 (29.1)	27 (37.0)	30 (27.3)	3 (13.0)	0.074^b^
Chronic kidney failure	45 (21.8)	10 (13.7)	28 (25.5)	7 (30.4)	0.085^c^
Epilepsy	36 (17.5)	19 (26.0)	12 (10.9)	5 (21.7)	0.022^c^
Chronic hepatic failure	35 (17.0)	9 (12.3)	23 (20.9)	3 (13.0)	0.300^c^
Stroke	34 (16.5)	15 (20.5)	17 (15.5)	2 (8.7)	0.416^c^
Neurosurgery	19 (9.2)	6 (8.2)	12 (10.9)	1 (4.3)	0.694^c^
Solid organ transplantation^£^	18 (8.7)	3 (3.1)	11 (10.0)	4 (17.4)	0.093^c^
Cerebral tumor	16 (7.8)	6 (8.2)	8 (7.3)	2 (8.7)	0.934^c^
Causes for altered state of consciousness					
General medical condition	126 (61.2)	45 (61.6)	72 (65.5)	9 (39.1)	0.060^b^
Acute CNS disease	54 (26.2)	16 (21.9)	31 (28.2)	7 (30.4)	0.034^b^
Post cardiac arrest	14 (6.8)	3 (4.1)	7 (6.4)	4 (17.4)	0.097^c^
Epilepsy	11 (5.3)	6 (8.2)	2 (1.8)	3 (13.0)	0.021^c^
Unknown	8 (3.9)	4 (5.5)	4 (3.6)	0 (0.0)	0.668^c^
Radiologic diagnosis^¥^					
Normal	16/165 (9.7)	5/52 (9.6)	9/93 (9.7)	2/20 (10.0)	1.000^c^
Acute structural damage^Ω^	52/165 (31.5)	14/52 (26.9)	30/93 (32.3)	8/20 (40.0)	0.565^b^
Chronic structural damage^€^	104/165 (63.0)	32/52 (61.5)	62/93 (66.7)	10/20 (50.0)	0.366^b^
Level of consciousness during first EEG					
Alert	35 (17.0)	14 (19.2)	16 (14.5)	5 (21.7)	0.514^c^
Obtundation	86 (41.7)	39 (53.4)	43 (39.1)	4 (17.4)	0.006^b^
Stupor	47 (22.8)	13 (17.8)	28 (25.5)	6 (26.1)	0.462^c^
Coma	25 (12.1)	6 (8.2)	15 (13.6)	4 (17.4)	0.371^c^
Sedated	13 (6.3)	1 (1.4)	8 (7.3)	4 (17.4)	0.020^c^

IP: interictal patterns; RPP: rhythmic and periodic patterns.

*p values were provided by (a) Kruskal-Wallis test, (b) Chi-squared test and (c) Fisher’s exact test.

Comparisons significant at the 0.016 level:

^#^: Ictal vs. IP

^&^: Ictal vs. RPP.

^£^: kidney and liver transplantation, CNS: central nervous system,

^¥^: radiologic diagnosis made by computed tomography or nuclear magnetic resonance image,

^Ω^: acute intracerebral, epidural or subdural lesions such as infarction, hemorrhage, CNS infectious disease or mass lesions,

^€^: cerebral atrophy or sequelae.

Ictal group patients were younger than IP and RPP group patients (Table 1). Previous history of epilepsy was less frequent in RPP group patients than in IP and Ictal groups (Table 1). General medical conditions were the main causes of consciousness alteration in all the groups studied [126/206 (61.2%) patients)], while acute CNS disease was observed in 54/206 (26.2%) patients, mainly CNS infection [14/54 (25.9%) patients] and acute ischemic stroke [13/54 (24.1%) patients] (Table 1).

A central nervous system image was performed in 165/206 (80.1%) patients (Table 1). The level of consciousness during the first EEG was similar between the three groups. Most patients were obtunded (41.7%) or stuporous (22.8%) during the first EEG recording. Only 25 out of 206 patients (12.1%) were in coma (Table 1).

### EEG analysis

Agreement between two raters (Interrater reliability) on EEG analysis was excellent (Kappa = 0.898; p<0.001). The main EEG findings are presented in Table 2.

**Table 2:** Main EEG findings. Values represent median (IQR) or No. (%).

Characteristics	All 475 (100.0)	IP 94 (19.8)	RPP 332 (69.9)	Ictal 49 (10.3)	P value*
Number of EEG	1 (1–2)	1 (1–1)	2 (1–4)^§^	1 (1–2)^#^	<0.001^a^
Interictal epileptiform discharges	322 (67.8)	50 (53.2)	232 (69.9)^§^	40 (81.6)^#^	0.001^b^
Triphasic waves	313 (65.9)	61 (64.9)	235 (70.8)	17 (34.7)^#^^&^	<0.001^b^
Generalized periodic discharges	117 (24.6)	0 (0.0)	105 (31.6)^§^	12 (24.5)^#^	<0.001^c^
Generalized periodic discharges +F	2 (0.4)	0 (0.0)	0 (0.0)	2 (4.1)^&^	0.010^c^
Generalized periodic discharges +R	10 (2.1)	0 (0.0)	10 (3.0)	0 (0.0)	0.142^c^
Lateralized periodic discharges	93 (19.6)	0 (0.0)	86 (25.9)^§^	7 (14.3)^#^	<0.001^c^
Lateralized periodic discharges +F	10 (2.1)	0 (0.0)	9 (2.7)	1 (2.0)	0.315^c^
Lateralized periodic discharges +R	8 (1.7)	0 (0.0)	7 (2.1)	1 (2.0)	0.421^c^
Bilateral periodic discharges	8 (1.7)	0 (0.0)	7 (2.1)	1 (2.0)	0.421^c^
Bilateral periodic discharges +F	0 (0.0)	0 (0.0)	0 (0.0)	0 (0.0)	
Bilateral periodic discharges +R	0 (0.0)	0 (0.0)	0 (0.0)	0 (0.0)	
Rithmic delta activity	60 (12.6)	0 (0.0)	59 (17.8)^§^	1 (2.0)^&^	<0.001^c^
Rithmic delta activity +S	11 (2.3)	0 (0.0)	11 (3.3)	0 (0.0)	0.111^c^
Spike and wave	4 (0.8)	0 (0.0)	4 (1.2)	0 (0.0)	0.729^c^
Eletrographic seizures	21 (4.4)	0 (0.0)	0 (0.0)	21 (42.8)	
Status epilepticus	11 (2.3)	0 (0.0)	0 (0.0)	11 (22.4)	

IP: interictal patterns, RPP: rhythmic and periodic patterns. +F: plus fast activity, +R: plus rhythmic activity, +S: plus sharp activity.

*p values were provided by (a) Kruskal-Wallis test, (b) Chi-squared test and (c) Fisher’s exact test.

Pairwise comparisons significant at the 0.016 level:

^#^: Ictal vs. IP,

^&^: Ictal vs. RPP

^§^: RPP vs. IP.

Interictal epileptiform discharges were observed in 322/475 (67.8%) EEGs and triphasic waves in 313/475 (65.9%) EEGs (Table 2). Interictal epileptiform discharges occurred in 40/49 (81.6%) EEGs from Ictal group and in 232/332 (69.9%) EEGs from RPP group, compared to 50/94 (53.2%) EEGs from IP group (p = 0.001) (Table 2). Triphasic waves were observed in 61/94 (64.9%) EEGs in IP group and 235/332 (69.9%) EEGs in RPP group, but only in 17/49 (34.7%) EEGs in Ictal group (p<0.001).

Generalized periodic discharges represented the most frequent periodic pattern in RPP and Ictal groups [117/475 (24.6%) EEGs], followed by lateralized periodic discharges [93/475 (19.6%) EEGs] (Table 2). Rhythmic delta activity was observed in 60/475 (12.6%) EEGs and spike-and-wave complexes in 4 (0.8%) of all EEGs, all in RPP group (Table 2).

Electrographic seizures occurred in 21 (42.8%) EEGs from 17 (73.9%) patients of Ictal group (Table 2 and Fig 1). Status epilepticus was observed in 11 (22.4%) EEGs from 10 (43.4%) patients. Furthermore, 13 (56.6%) patients in Ictal group presented some additional periodic pattern on EEGs, and one (4.3%) patient had RDA on EEG (Fig 1).

In RPP group, 80/110 (72.7%) patients showed some periodic pattern in a total of 154 EEGs (Fig 1). Out of those, 22 (20%) patients presented EEGs (43 EEGs, 12.9%) with RDA and 1 (0.9%) patient showed spike and wave (SW) (3 EEGs, 0.9%) (Fig 1). A combination of PDs and RDA occurred in 16 (4.8%) EEGs out of 12 (10.9%) patients and one patient had PD and SW in the same EEG (Fig 1). These pattern combinations were not observed in other groups.

### Treatment

Treatment with AEDs, IVADs or a combination of both was administered in 102/204 (50%) patients (Table 3). While all patients in Ictal group received some treatment (AEDs or IVADs), only 24/73 (32.9%) patients in IP group and 55/108 (50.9%) patients in RPP group were treated (p<0.001) (Table 3).

**Table 3:** Antiepileptic treatment. Values represent median (IQR) or No. /Total No. (%).

Characteristics	All N:206 (100.0)	IP N: 73 (35.4)	RPP N:110 (53.4)	Ictal N:23 (11.2)	P value*
Received treatment	102/204 (50.0)	24/73 (32.9)	55/108 (50.9)^§^	23/23 (100.0)^#^^&^	<0.001^a^
Antiepileptic drugs	100/204 (49.0)	24/73 (32.9)	53/108 (49.1)	23/23 (100.0)^#^^&^	<0.001^a^
Anaesthesic drugs	32/204 (15.7)	1/73 (1.4)	19/108 (17.6)^§^	12/23 (52.2)^#^^&^	<0.001^a^
Duration of antiepileptic treatment (days)	13 (6–34)	8 (5–24)	19 (9–43)	10 (5–15)^$^	0.020^b^
Duration of anesthetic treatment (days)	3 (2–7)	5 (5–5)	3 (2–7)	3 (3–11)	0.807^b^

IP: interictal patterns, RPP: rhythmic and periodic patterns.

*p values were provided by (a) Chi-squared test and (b) Kruskal-Wallis test.

Pairwise comparisons significant at the 0.016 level:

^#^: Ictal vs. IP,

^&^: Ictal vs. RPP

^§^: RPP vs. IP.

Pairwise comparisons significant at the 0.025 level:

^$^: Ictal vs. RPP.

Antiepileptic drugs were administered to 100/204 (49%) patients, and IVADs to 32/204 (15.7%) patients (Table 3). Patients in all groups received AEDs: 24/73 (32.9%) in IP group, 53/108 (49.1%) in RPP group and all patients in Ictal group (p<0.001; Table 3).

### Outcomes

**Fig 5:**
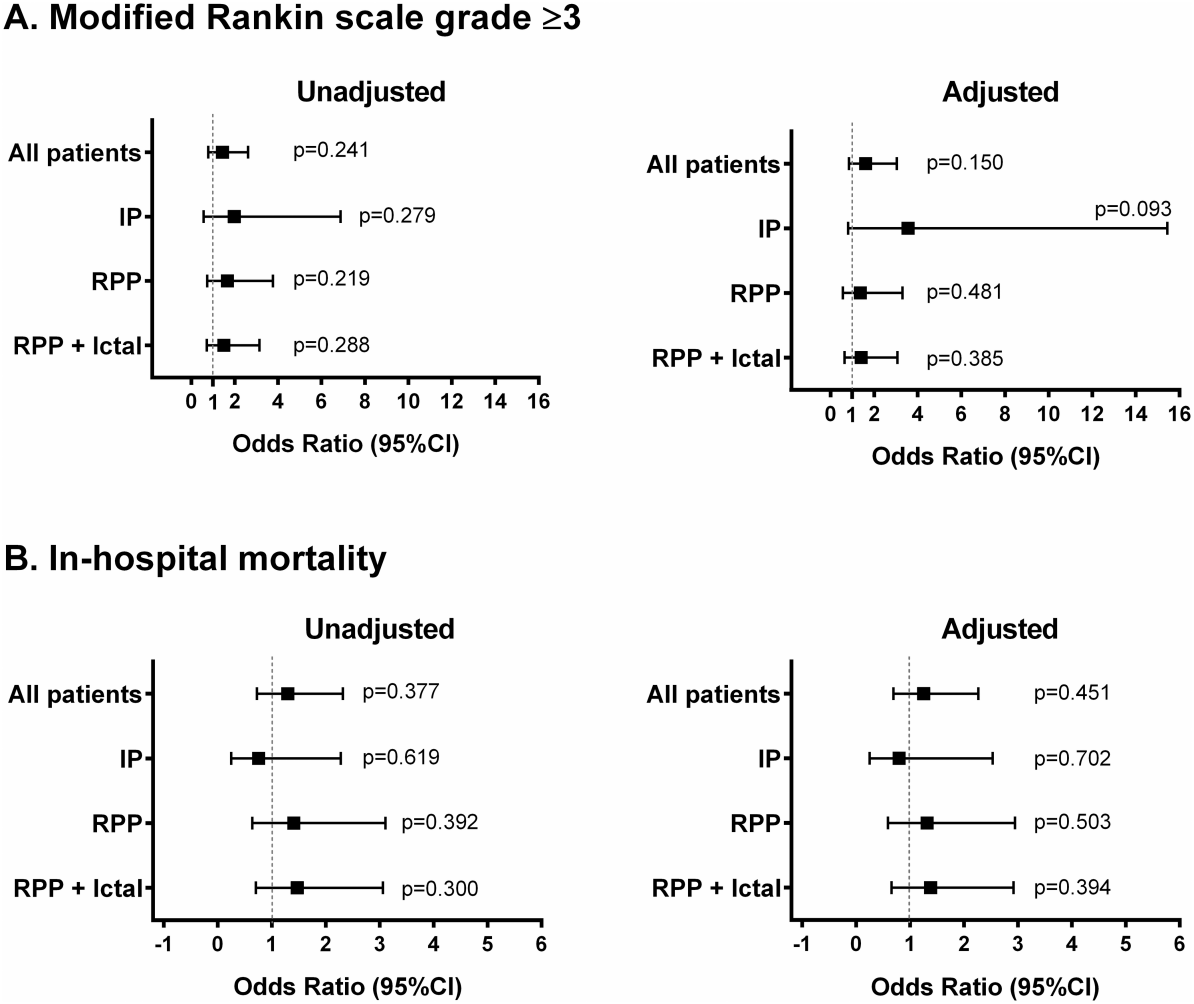
Effect of antiepileptic treatment on unfavorable outcomes in all patients and accordingly to study groups. IP: interictal patterns, RPP: rhythmic and periodic patterns group, CI: confidence interval. An unfavorable outcome was defined as Modified Rankin scale grade ≥3 (Panel A) or in-hospital mortality (Panel B).

Out of 206 patients, 145 (70.4%) were referred for ICU admission (Table 4). Ictal and RPP patients were more frequently admitted to the ICU than IP group patients (Table 4). However, ICU and hospital LOS did not differ between the groups (Table 4). A Modified Rankin scale ≥3 at hospital discharge [141/203 (69.5%) patients] and in-hospital mortality [71/206 (34.5%) patients] did not differ between the three groups (Table 4). Antiepileptic treatment did not affect the risk of unfavorable outcomes (modified Rankin scale ≥3 and in-hospital mortality) in a crude and adjusted logistic regression analysis in all the groups (Fig 5).

**Table 4:** Study outcomes. Values represent median (IQR) or No. /Total No. (%).

Characteristics	All N:206 (100)	IP N: 73 (35.4)	RPP N:110 (53.4)	Ictal N:23 (11.2)	P value*
ICU admission	145/206 (70.4)	42/73 (57.5)	82/110 (74.5)^§^	21/23 (91.3)^#^^&^	0.003^a^
Mechanical ventilation	83/206 (40.3)	23/73 (31.5)	48/110 (43.6)	12/23 (52.2)	0.124^a^
Duration of mechanical ventilation (days)	8 (3–12)	5 (3–11)	9 (4–14)	6 (3–12)	0.371^b^
ICU length of stay (days)	11 (4–23)	9 (5–25)	13 (5–24)	11 (3–14)	0.597^b^
Hospital length of stay (days)	23 (11–56)	21 (6–45)	28 (13–74)	15 (8–43)	0.070^b^
In-hospital mortality	71/206 (34.5)	21/73 (28.8)	40/110 (36.4)	10/23 (43.5)	0.377^c^
Dichotomized modified Rankin scale					
Favorable outcome	62/203 (30.5)	19/71 (26.8)	35/109 (32.1)	8/23 (34.8)	0.670^a^
Unfavorable outcome	141/203 (69.5)	52/71 (73.2)	74/109 (67.9)	15/23 (65.2)	

IP: interictal patterns, RPP: rhythmic and periodic patterns. A “favorable” outcome was defined as Modified Rankin scale grade ≤2.

*p values were provided by (a) Chi-squared test, (b) Kruskal-Wallis test and (c) Fisher’s exact test.

Pairwise comparisons significant at the 0.016 level:

^#^: Ictal vs. IP,

^&^: Ictal vs. RPP

^§^: RPP vs. IP.

The need for mechanical ventilation, ICU and hospital LOS, in-hospital mortality, and frequency of Modified Rankin scale ≥3 at hospital discharge did not differ between treated and non-treated IP group patients (S1 Table).

In RPP group, treated patients were more frequently mechanically ventilated than non-treated patients [31/55 (56.4%) vs. 16/53 (30.2%), respectively for treated and non-treated patients, p = 0.006)] (S2 Table). We observed a higher intubation rate in IVADs treated patients than AEDs exclusively treated patients [16/19 (84.2%) vs. 15/36 (41.7%) patients, respectively for IVADs and AEDs, p = 0.002] (S3 Table). Treated and non-treated RPP patients had similar ICU and hospital LOS, whether they received IVADs or AEDs as treatment (S2 and S3 Tables). In-hospital mortality did not differ between treated and non-treated RPP patients (S2 Table). However, in-hospital mortality was higher in IVADs treated patients compared to AEDs treated patients [11/19 (57.9%) vs. 11/36 (30.6%) patients, respectively for IVADs and AEDs, p = 0.049] (S3 Table).

Out of 23 Ictal group patients, 11 (47.8%) received only AEDs and 12 (52.2%) had IVADs associated treatment (S4 Table). The need for mechanical ventilation, ICU and hospital LOS, in-hospital mortality, and frequency of Modified Rankin scale ≥3 at hospital discharge did not differ between Ictal patients treated exclusively with AEDs or IVADs (S4 Table).

## Discussion

In this study, we investigated the effect of antiepileptic treatment on outcomes in encephalopathic patients with different patterns of EEG. We found that antiepileptic treatment was not associated with reduced risk of unfavorable outcome or death in patients with impaired consciousness and altered EEG. The lack of antiepileptic treatment benefit was observed regardless the age, encephalopathy etiology, and EEG patterns. These results suggest that treatment responses depend on the likelihood of neuronal injury from each pattern in a given clinical setting.

We included patients with impairment consciousness ranging from mental confusion to coma. Multiple causes were associated, and more than one etiology could be coexisting in a given patient. However, after classifying patients in predetermined groups, etiologies were proportionally distributed. Most patients had some general medical condition related to encephalopathy. Metabolic disorders were the most common cause, observed in 77% of patients. Acute CNS disease occurred in 26% of patients, mainly CNS infection and acute ischemia. This heterogeneity of etiologies agrees with the wide range of neurological impairment observed. As expected, EEG patterns were heterogeneous as well.

In a similar study of routine-EEG in encephalopathic patients, Koren et al. investigated 655 EEGs from 371 critically ill patients and found approximately 84% of normal or clearly interictal patterns, 12% of patterns authors called ‘ictal–interictal uncertainty’ patterns (RPPIIIU) and 4% of electrographic seizures [26]. Contrary to our study, the authors included only patients with acute seizures or clinical suspicion of NCS or NCSE [26]. The authors selected patients with Glasgow Coma Scale ranging from 3 to 15 allocated in all three groups of EEG patterns [26]. Investigators did not assess treatment information. They concluded that RPPIIIU (that correlate with RPP pattern in our study) were highly predictive for NCS and should trigger a continuous EEG monitoring, since 20% of all patients with those patterns also showed electrographic seizures, versus only 0.9% of other patients [26]. In our cohort, approximately 56% of Ictal group patients had some periodic patterns coexisting in the same routine EEG, agreeing with the close relation of periodic patterns and NC seizures.

In a study to assess predictive variables of status prognostic, Rossetti and cols focused on the variables available in the hospital presentation [27]. They found that old age and marked impairment of consciousness were predictive of death, although underlying “acute symptomatic etiologies” rather than status per se, seemed to be the main determinant of outcome [27]. As in most status epilepticus studies, the researchers excluded anoxic-ischemic patients. “Acute symptomatic etiology” was a heterogeneous group, including SE related to drug withdrawal, which usually has a better outcome, and CNS tumors or encephalitis, called “potentially fatal etiologies” in the study. Specific causes of SE were primary CNS disease, mostly stroke, tumor or CNS infection. Metabolic disorders accounted for fewer cases of status [27]. In our study, we included post anoxic patients. Nonconvulsive status epilepticus was related to acute medical condition in most cases, especially metabolic disorders, and we had few cases of acute neurologic etiology. The selection of patients must have played a role: Rossetti and cols selected patients in an EEG data bank, and we looked for patients with altered consciousness who had an EEG performed [27].

In our study, all Ictal group patients received AEDs, for a median period of 10 days. Out of 23 patients, 12 (52.2%) received IVADs, for a median of 3 days. Although treated more frequently with IVADs, Ictal group patients did not need more intubation, or spent more days on mechanical ventilation. In this group, the use of IVADs did not affect ICU and hospital LOS or in-hospital mortality. The fact that treating an EEG showing seizures or status epilepticus was not associated with better outcomes even when treating more aggressively with IVADs, agrees with most clinical trials that have concluded that SE etiology is the main determinant of outcome [27,28].

This study have some limitations. The analysis based only on routine EEGs implies in a shorter monitoring period, and it is possible that continuous EEG monitoring would allocate patients in different groups. However, EEG monitoring is not available in most ICUs worldwide. Besides, we intent to assess the main EEG features of patients with acute consciousness alteration and investigate if those patterns could provide reliable information on prognostication and treatment decision. Finally, the small sample size of Ictal group (23 patients) precluded us to evaluate the impact of AEDs and IVADs treatment on clinical outcomes.

## Conclusion

In patients with acute altered consciousness and abnormal routine electroencephalogram, treatment with antiepileptic drugs or intravenous anesthetic drugs did not improve the incidence of unfavorable outcomes, regardless of the presence of periodic, rhythmic or ictal electroencephalogram patterns. The effect of intravenous anesthetic drugs on hospital mortality in RPP patients should be further verified in prospective, controlled, clinical studies.

## Supporting information

S1 Table Study outcomes of interictal patterns patients according to the treatment status. Values represent median (IQR) or No. /Total No. (%). An unfavorable outcome was defined as Modified Rankin scale grade ≥3. *p values were provided by (a) Chi-squared test and (b) Mann-Whitney U test.
(DOCX)

S2 Table Study outcomes of rhythmic and periodic patterns patients according to the treatment status. Values represent median (IQR) or No. /Total No. (%). An unfavorable outcome was defined as Modified Rankin scale grade ≥3. * p values were provided by (a) Chi-squared test and (b) Mann-Whitney U test. ^#^: Two patients with unknown treatment status.
(DOCX)

S3 Table Study outcomes of rhythmic and periodic patterns patients according to the treatment received. Values represent median (IQR) or No. /Total No. (%). An unfavorable outcome was defined as Modified Rankin scale grade ≥3. * p values were provided by (a) Chi-squared test and (b) Mann-Whitney U test. ^#^: Two patients with unknown treatment status.
(DOCX)

S4 Table Study outcomes of ictal patients according to the treatment received. Values represent median (IQR) or No. /Total No. (%). An unfavorable outcome was defined as Modified Rankin scale grade ≥3. *p values were provided by (a) Fisher exact test and (b) Mann-Whitney U test.
(DOCX)
