# Supplementary material for: Outcomes of patients with altered level of consciousness and abnormal electroencephalogram: A retrospective cohort study
Source: PLoS One. 2017 Sep 8;12(9):e0184050. doi: 10.1371/journal.pone.0184050 (PMC5590878; doi:10.1371/journal.pone.0184050)
Supplement: S1 Table — Values represent median (IQR) or No. /Total No. (%). An unfavorable outcome was defined as Modified Rankin scale grade ≥3. *p values were provided by (a) Chi-squared test and (b) Mann-Whitney U test. (DOCX) [file pone.0184050.s001.docx]

**S1 Table.** Study outcomes of interictal patterns patients according to the treatment status. Values represent median (IQR) or No./Total No. (%).

| **Characteristics** | **Non-treated**  **N= 49/73**  **(67.1)** | **Treated**  **N= 24/73 (32.9)** | **P value*** |
| --- | --- | --- | --- |
| Mechanical ventilation | 14/49 (28.6) | 9/24 (37.5) | 0.440^a^ |
| Duration of mechanical ventilation (days) | 5 (3 - 14) | 8 (4 - 10) | 0.748^b^ |
| Length of ICU stay (days) | 9 (4 - 25) | 8 (7 - 15) | 0.661^b^ |
| Length of hospital stay (days) | 21 (7 - 49) | 21 (6 - 43) | 0.626^b^ |
| In-hospital mortality | 15/49 (30.6) | 6/24 (25.0) | 0.619^a^ |
| Dichotomized modified Rankin scale |  |  | 0.274^a^ |
| Favorable outcome | 15/49 (30.6) | 4/22 (18.2) |  |
| Unfavorable outcome | 34/49 (69.4) | 18/22 (81.8) |  |

An unfavorable outcome was defined as Modified Rankin scale grade ≥3. *p values were provided by (a) Chi-squared test and (b) Mann-Whitney U test.
