# Supplementary material for: Outcomes of patients with altered level of consciousness and abnormal electroencephalogram: A retrospective cohort study
Source: PLoS One. 2017 Sep 8;12(9):e0184050. doi: 10.1371/journal.pone.0184050 (PMC5590878; doi:10.1371/journal.pone.0184050)
Supplement: S2 Table — Values represent median (IQR) or No. /Total No. (%). An unfavorable outcome was defined as Modified Rankin scale grade ≥3. * p values were provided by (a) Chi-squared test and (b) Mann-Whitney U test. #: Two patients with unknown treatment status. (DOCX) [file pone.0184050.s002.docx]

**S2 Table.** Study outcomes of rhythmic and periodic patterns patients according to the treatment status. Values represent median (IQR) or No./Total No. (%).

| **Characteristics^#^** | **Non-treated**  **N= 53/108 (49.1)** | **Treated**  **N= 55/108 (50.9)** | **P value*** |
| --- | --- | --- | --- |
| Mechanical ventilation | 16/53 (30.2) | 31/55 (56.4) | 0.006^a^ |
| Duration of mechanical ventilation (days) | 9 (5-14) | 10 (4-14) | 0.881^b^ |
| Length of ICU stay (days) | 12 (3-19) | 14 (6-29) | 0.133^b^ |
| Length of hospital stay (days) | 27 (12-67) | 29 (14-66) | 0.531^b^ |
| In-hospital mortality | 17/53 (32.1) | 22/55 (40.0) | 0.391^a^ |
| Dichotomized modified Rankin scale |  |  |  |
| Favorable outcome | 20/52 (38.5) | 15/55 (27.3) | 0.218^a^ |
| Unfavorable outcome | 32/52 (61.5) | 40/55 (72.7) |  |

An unfavorable outcome was defined as Modified Rankin scale grade ≥3. * p values were provided by (a) Chi-squared test and (b) Mann-Whitney U test. ^#^: Two patients with unknown treatment status.
