# Supplementary material for: Outcomes of patients with altered level of consciousness and abnormal electroencephalogram: A retrospective cohort study
Source: PLoS One. 2017 Sep 8;12(9):e0184050. doi: 10.1371/journal.pone.0184050 (PMC5590878; doi:10.1371/journal.pone.0184050)
Supplement: S3 Table — Values represent median (IQR) or No. /Total No. (%). An unfavorable outcome was defined as Modified Rankin scale grade ≥3. * p values were provided by (a) Chi-squared test and (b) Mann-Whitney U test. #: Two patients with unknown treatment status. (DOCX) [file pone.0184050.s003.docx]

**S3 Table.** Study outcomes of rhythmic and periodic patterns patients according to the treatment received. Values represent median (IQR) or No./Total No. (%).

| **Characteristics^#^** | **Antiepileptic drugs**  **N= 36/55 (65.5)** | **Anesthetic drugs**  **N= 19/55 (34.5)** | **P value*** |
| --- | --- | --- | --- |
| Mechanical ventilation | 15/36 (41.7) | 16/19 (84.2) | 0.002^a^ |
| Duration of mechanical ventilation (days) | 7 (2-11) | 11 (7-16) | 0.169^b^ |
| Length of ICU stay (days) | 11 (4-29) | 18 (7-30) | 0.115^b^ |
| Length of hospital stay (days) | 31 (13-87) | 25 (21-50) | 0.915^b^ |
| In-hospital mortality | 11/36 (30.6) | 11/19 (57.9) | 0.049^a^ |
| Dichotomized modified Rankin scale |  |  |  |
| Favorable outcome | 11/36 (30.6) | 4/19 (21.1) | 0.452^a^ |
| Unfavorable outcome | 25/36 (69.4) | 15/19 (78.9) |  |

An unfavorable outcome was defined as Modified Rankin scale grade ≥3. * p values were provided by (a) Chi-squared test and (b) Mann-Whitney U test. ^#^: Two patients with unknown treatment status.
