# Supplementary material for: Outcomes of patients with altered level of consciousness and abnormal electroencephalogram: A retrospective cohort study
Source: PLoS One. 2017 Sep 8;12(9):e0184050. doi: 10.1371/journal.pone.0184050 (PMC5590878; doi:10.1371/journal.pone.0184050)
Supplement: S4 Table — Values represent median (IQR) or No. /Total No. (%). An unfavorable outcome was defined as Modified Rankin scale grade ≥3. *p values were provided by (a) Fisher exact test and (b) Mann-Whitney U test. (DOCX) [file pone.0184050.s004.docx]

**S4 Table.** Study outcomes of ictal patients according to the treatment received. Values represent median (IQR) or No./Total No. (%).

| **Characteristics** | **Antiepileptic drugs**  **N= 11/23 (47.8)** | **Anesthetic drugs**  **N= 12/23 (52.2)** | **P value*** |
| --- | --- | --- | --- |
| Mechanical ventilation | 4/11 (36.4) | 8/12 (66.7) | 0.220^a^ |
| Duration of mechanical ventilation (days) | 4 (3-75) | 7 (3-12) | 0.836^b^ |
| Length of ICU stay (days) | 13 (3-47) | 8 (3-13) | 0.337^b^ |
| Length of hospital stay (days) | 22 (6-75) | 15 (10-22) | 0.478^b^ |
| In-hospital mortality | 3/11 (27.3) | 7/12 (58.3) | 0.214^a^ |
| Dichotomized modified Rankin scale |  |  |  |
| Favorable outcome | 6/11 (54.5) | 2/12 (16.7) | 0.089^a^ |
| Unfavorable outcome | 5/11 (45.5) | 10/12 (83.3) |  |

An unfavorable outcome was defined as Modified Rankin scale grade ≥3. *p values were provided by (a) Fisher exact test and (b) Mann-Whitney U test.
